# Systemische Entzündung, „Sickness Behavior“ und Erwartungsprozesse: Welche Rolle spielen Erwartungen bei entzündungsassoziierten Symptomen?

**DOI:** 10.1007/s00482-021-00602-0

**Published:** 2021-10-29

**Authors:** Justine Schmidt, Johanna Reinold, Regine Klinger, Sven Benson

**Affiliations:** 1 Institut für Medizinische Psychologie und Verhaltensimmunbiologie, Zentrum für Translationale Neuro- und Verhaltenswissenschaften, Universitätsklinikum Essen, Med. Fakultät, Universität Duisburg-Essen, Hufelandstraße 55, 45122 Essen, Deutschland; 2 Klinik für Infektiologie, Universitätsklinikum Essen, Universität Duisburg-Essen, Essen, Deutschland; 3 Klinik und Poliklinik für Anästhesiologie, Universitätsklinikum Hamburg Eppendorf, Universität Hamburg, Hamburg, Deutschland

**Keywords:** Schmerz, Endotoxämie, Zytokine, Chronische Erkrankung, Emotionen, Pain, Endotoxemia, Cytokines, Chronic disease, Emotions

## Abstract

**Hintergrund:**

Systemische Entzündungsprozesse gehen mit unspezifischen körperlichen und psychischen Krankheitssymptomen einher, darunter Schmerz und affektbezogene Symptome. Diese immunvermittelten Symptome („Sickness Behavior“) beruhen auf der zentralnervösen Wirkung von Immunbotenstoffen wie proinflammatorischen Zytokinen und vermitteln bei akuten Entzündungsreaktionen, etwa nach einer Impfung oder Verletzung, ein adaptives Schonverhalten. Bei chronischen Entzündungsprozessen können die Symptome des Sickness Behavior jedoch zu Einschränkungen der Lebensqualität führen und zur Komorbidität bei chronischen Schmerzerkrankungen beitragen. Trotz der hohen klinischen Relevanz des Sickness Behavior wurden bisher psychologische Ansätze zur Modulation der immunvermittelten Sickness-Symptome kaum untersucht. Einen Ansatz könnte die Nutzung von Erwartungseffekten bieten, da positive und negative Erwartungen (Placebo- bzw. Nocebo-Effekte) nachweislich einen Einfluss auf Schmerz und affektbezogene Symptome haben.

**Ziel der Arbeit:**

In dieser Übersichtsarbeit werden die immunologischen und psychobiologischen Faktoren, die zu Schmerz im Kontext des Sickness Behavior beitragen, zusammengefasst. Aufbauend wird diskutiert, wie durch positive und negative Erwartungen Sickness-Symptome beeinflusst werden können und welche biologischen und psychologischen Mechanismen dabei involviert sind. Ziel ist es, potenzielle Ansatzpunkte zur Optimierung von Erwartungen im Kontext immunvermittelter Sickness-Symptome zu identifizieren. Perspektivisch lassen sich darauf aufbauend Interventionen entwickeln, um diese Symptome zu reduzieren sowie die Wirkungen und Nebenwirkungen von immunassoziierten Therapien durch gezielte Erwartungsinduktionen im Rahmen der Kommunikation mit Patient:innen positiv zu beeinflussen.

## Abstract (en)

**Background:**

Systemic inflammation is accompanied by unspecific physical and psychological symptoms of sickness, including pain and affective symptoms. These symptoms (commonly called “sickness behavior”) are mediated by the central nervous effects of immune messengers such as pro-inflammatory cytokines. While adaptive during acute inflammation, sickness symptoms can have detrimental effects on quality of life during chronic inflammation and may contribute to comorbidity in chronic pain conditions. Despite the high clinical relevance of sickness behavior, psychological interventions aiming to modulate sickness symptoms have hardly been investigated. One approach could be the use of expectation effects, since positive and negative expectations (placebo or nocebo effects) have been shown to have an influence on pain and affect-related symptoms.

**Objectives:**

Herein, we summarize immunological and psychobiological factors that contribute to pain in the context of sickness behavior, with a major focus on findings from experimental endotoxemia. Against this background, we discuss how expectations could help to improve immune-mediated sickness symptoms and outline potential psychological and psychobiological mechanisms underlying this putative effect.

## Hintergrund

Systemische Entzündungsprozesse gehen mit Krankheitssymptomen wie z. B. Schmerz einher. Insbesondere bei chronischen Entzündungsprozessen können diese immunvermittelten Symptome eine erhebliche Belastung für Betroffene darstellen. Weitgehend unklar ist, inwieweit solche Symptome durch psychologische Interventionen gelindert werden können. Anhand von Befunden aus der Entzündungs- und Placeboforschung soll hier diskutiert werden, wie sich immunvermittelte Krankheitssymptome und auch die Nebenwirkungen von immunassoziierten Therapien durch die Optimierung von Erwartungsprozessen und ärztliche Kommunikation beeinflussen lassen.

## Systemische Entzündung und Sickness Behavior: Immunsystem, Psyche und Schmerz

**Figure 1:**
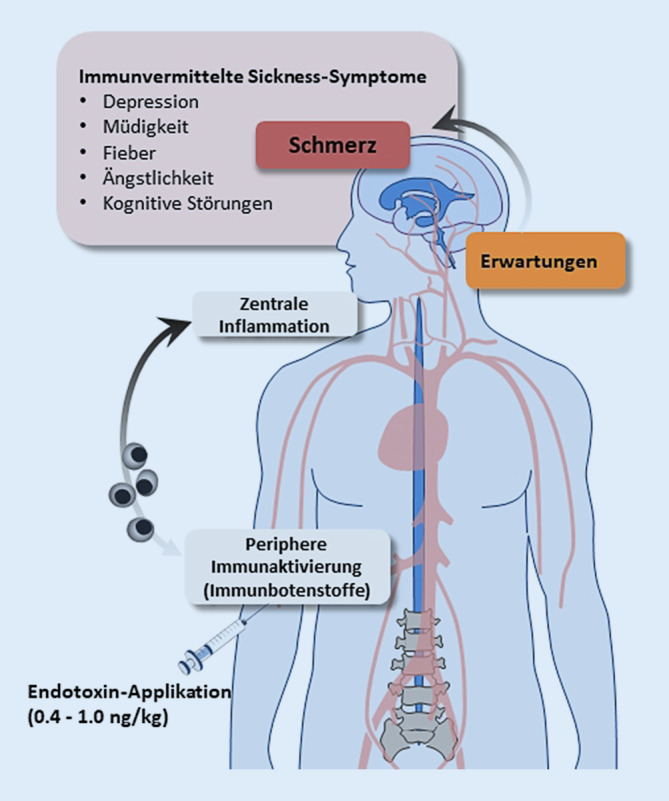

Das Immunsystem und das zentrale Nervensystem stehen in einem engen und kontinuierlichen Informationsaustausch. Dadurch können einerseits Immunprozesse an Umweltbedingungen, physiologische Stressoren oder psychologische Belastungen angepasst werden. Auf der anderen Seite erhält das Gehirn über afferente Kommunikationswege Informationen über den Status des Immunsystems [10, 25]. Dies erlaubt es, das Verhalten an eine Krankheitssituation anzupassen, etwa im Falle eines akuten Infekts oder bei einer entzündlichen Erkrankung. Hierzu steuern Immunbotenstoffe wie die pro-inflammatorischen Zytokine TNF‑α und Interleukin(IL)-6 nicht nur die lokale und systemische Immunantwort. Zytokine können auch die Aktivität des zentralen Nervensystems (ZNS) über unterschiedliche humorale und neurale Kommunikationswege wie den afferenten Vagusnerv beeinflussen. In der Folge kommt es zu Veränderungen neuroendokriner und metabolischer Prozesse sowie Veränderungen des Verhaltens und Befindens, die unter dem Begriff des „Sickness Behavior“ oder „Sickness Syndrome“ zusammengefasst werden ([10]; Abb. 1). Beim immunvermittelten Sickness Behavior treten verschiedene unspezifische Krankheitssymptome auf körperlicher und psychischer Ebene in Erscheinung, darunter eine erhöhte Schmerzsensitivität, Dysthymie und Ängstlichkeit, Fatigue, Veränderungen von Schlaf und Appetit sowie leichte kognitive Beeinträchtigungen [10, 12, 25]. Im Falle einer akuten Entzündungsreaktion – etwa nach Gewebeschädigung bei Verletzungen oder operativen Eingriffen, bei akuten Infektionen oder nach einer Impfung – sind diese Symptome als eine zwar unangenehme, aber adaptive Reaktion unseres Körpers zu verstehen, da sie zu Schonung, sinnvoller Ressourcenallokation und zur Rekonvaleszenz beitragen. Bei chronischen oder rezidivierenden Entzündungsprozessen können die Symptome jedoch die körperliche und psychische Funktionsfähigkeit und die gesundheitsbezogene Lebensqualität deutlich beeinträchtigen [10, 19]. Außer bei chronisch-entzündlichen und Autoimmunerkrankungen treten immunvermittelte Sickness-Symptome auch als Nebenwirkung von Zytokin- oder Chemotherapien auf [10, 16]. Im Kontext von Schmerzerkrankungen können Entzündungsprozesse sowohl die Schmerzsensitivität und das Chronifizierungsrisiko erhöhen als auch komorbid auftretende affektive Störungen und weitere unspezifische Krankheitssymptome begünstigen [12, 16, 30].

Trotz der weiten Verbreitung des Sickness Behavior und der resultierenden Belastung für Betroffene sind psychologische Interventionen zur Reduktion dieser immunvermittelten Symptome bisher kaum untersucht worden. Im Folgenden sollen daher die psychobiologischen Faktoren, die insbesondere zu Schmerz als Symptom des Sickness Behavior beitragen, zunächst anhand experimenteller Befunde aus der Entzündungsforschung zusammengefasst werden, um so potenzielle Ansatzpunkte für die Entwicklung und Nutzung von psychologisch orientierten Interventionen zur Optimierung von Erwartungsprozessen aufzuzeigen (Abb. 1).

## Schmerz als Symptom des Sickness Behavior

Entzündungsmediatoren wie proinflammatorische Zytokine tragen auf lokaler Ebene zu einer Aktivierung von Nozizeptoren sowie auf Ebene des ZNS zu einer zentralen Sensitivierung bei [12, 23]. Klinische Studien unterstützen die Bedeutung systemischer Entzündungsprozesse und einer veränderten Neuro-Immun-Kommunikation für die Pathophysiologie chronisch-entzündlicher und potenziell auch funktioneller Schmerzerkrankungen [12, 30]. Jedoch lässt sich anhand der oftmals korrelativen Befunde für diese Erkrankungen nicht eindeutig klären, ob erhöhte Zytokinspiegel eine Ursache der Schmerzsymptomatik sind oder vielmehr eine Folge der psychischen Belastung bei chronischen Schmerzen darstellen. Daher wird in Ergänzung zu tierexperimentellen Ansätzen seit einigen Jahren das Modell der experimentellen Endotoxämie eingesetzt, um die Effekte von systemischen Entzündungsprozessen auf die Schmerzwahrnehmung und -bewertung sowie auf die zentrale Schmerzverarbeitung unter kontrollierten, standardisierten Bedingungen beim Menschen zu untersuchen [2, 28]. Hierzu wird in niedrigen Dosierungen bakterielles Endotoxin wie Lipopolysaccharid (LPS) appliziert, welches als prototypisches pathogen-assoziiertes Molekülmuster über Toll-like-Rezeptor‑4 (TLR-4)-abhängige Pfade zur Synthese und Freisetzung von proinflammatorischen Zytokinen führt [2]. Durch die intravenöse Injektion von isoliertem LPS lässt sich somit eine transiente systemische Immunaktivierung induzieren (zusammenfassend s. [25, 28]). Hierbei sind im Zeitraum von ca. 1–4 h nach Injektion dosisabhängige Anstiege proinflammatorischer Zytokine wie TNF‑α und IL‑6 sowie neuroendokriner Mediatoren wie Cortisol zu beobachten. Diese Effekte werden von grippeähnlichen Krankheitssymptomen und einer beeinträchtigten, negativen (depressionsähnlichen) Stimmungslage begleitet [5, 17]. Durch die Nutzung der funktionellen Magnetresonanztomografie (fMRT) und die Analyse von Cerebrospinalflüssigkeit (CSF) nach LPS-Gabe wurde dokumentiert, dass die ausgelösten peripheren Entzündungsprozesse zu transienten Reaktionen auf der Ebene des ZNS führen, welche Erklärungsansätze für die Entstehung des Sickness Behavior bieten [17, 25].

## Befunde der experimentellen Endotoxämie: Entzündung und Schmerz

Das Modell der experimentellen Endotoxämie wurde inzwischen in mehreren randomisierten, Placebo-kontrollierten Studien eingesetzt, um Veränderungen der Schmerzsensitivität während einer systemischen Entzündungsreaktion bei gesunden Testpersonen zu untersuchen. Besonders gut dokumentiert ist hierbei eine Abnahme der Druckschmerzschwellen, welche für unterschiedliche Muskelgruppen des Körpers gezeigt wurde [8, 15, 31, 32]. Dieser Effekt tritt zum Zeitpunkt erhöhter Plasmazytokinspiegel auf, erscheint dosisabhängig zu erfolgen und ist mit der Konzentration proinflammatorischer Zytokine im Plasma korreliert [8, 31]. Neben einer Abnahme von muskuloskeletalen Druckschmerzschwellen wurden reduzierte Wahrnehmungs- und Schmerzschwellen für rektale Distensionsreize gezeigt [9, 32], sodass von einem LPS-vermittelten Effekt insbesondere auf die Tiefensensibilität auszugehen ist [31]. Auf zentraler Ebene wurde mittels fMRT gezeigt, dass während einer LPS-induzierten systemischen Immunaktivierung die neurale Verarbeitung von viszeralen [9] bzw. Druckschmerzreizen [14] verändert ist. Neben einer erhöhten Sensitivität gegenüber experimentell applizierten Schmerzreizen treten nach LPS-Gabe auch spontane, grippetypische Schmerzsymptome wie Kopf‑, Muskel- und Gelenkschmerzen auf [7].

Eine ebenfalls gut dokumentierte Folge der LPS-induzierten systemischen Immunaktivierung ist das vorübergehende Auftreten von Stimmungsbeeinträchtigungen im Sinne einer negativen, depressionsähnlichen bzw. ängstlich gefärbten Stimmung [17]. Vor dem Hintergrund der engen und reziproken Verbindung von Schmerz und negativen Emotionen [30] stellt sich die Frage, inwieweit eine veränderte Schmerzsensitivität während einer systemischen Entzündungsreaktion durch negative Emotionen (mit) verursacht wird. So weisen mehrere Endotoxin-Studien darauf hin, dass eine erhöhte Schmerzsensitivität nach LPS-Gabe sowohl mit dem Anstieg von proinflammatorischen Zytokinen als auch mit der LPS-vermittelten negativen Stimmung assoziiert ist. Dieser Effekt wird vermutlich durch (Risiko‑)Faktoren wie psychologischen Distress weiter verstärkt [7].

Zusammenfassend unterstützen die dargestellten experimentellen Befunde, dass systemische Entzündungsprozesse nicht nur zu einer erhöhten Schmerzsensitivität beitragen, sondern auch zu einer Beeinträchtigung der Stimmung, was von Bedeutung für komorbid auftretende affektive Störungen bei chronischen Schmerzerkrankungen sein kann. Darüber hinaus bieten die beschriebenen Prozesse einen Erklärungsansatz für das Auftreten unspezifischer Krankheitssymptome im Kontext von Entzündungsprozessen, wie sie etwa nach akuten Verletzungen oder operativen Eingriffen sowie bei chronischen Schmerzerkrankungen beobachtet werden. Die experimentelle Endotoxämie bietet somit einen interessanten Ansatz, um unter kontrollierten laborexperimentellen Bedingungen immunvermittelte Sickness-Symptome zu analysieren und darauf aufbauend innovative Therapieansätze zu entwickeln [28].

## Können Erwartungsprozesse die Symptome des Sickness Behavior beeinflussen?

Patient:innen entwickeln Erwartungen zum Verlauf von Erkrankungen und zum Erfolg von Therapien aufgrund von Informationen, die beispielsweise im ärztlichen Gespräch oder durch Medien vermittelt werden, sowie auf der Basis von Lernprozessen und Erfahrungen. Solche positiven (und auch negativen) Erwartungen beeinflussen die Wahrnehmung und Bewertung von Symptomen und können darüber hinaus auch die Effekte von therapeutischen und pharmakologischen Interventionen beeinflussen. Erwartungseffekte wurden eindrucksvoll für akute und chronische Schmerzen (s. die weiteren Beiträge in diesem Sonderheft) sowie auch im Kontext affektiver Erkrankungen beschrieben [21, 24, 29]. Dementsprechend könnten positive Erwartungen zu einer Verbesserung von immunvermittelten Sickness-Symptomen oder einer besseren Wirkung von entzündungshemmenden Medikamenten beitragen, negative Erwartungen hingegen zu einer stärkeren Symptomatik oder unerwünschten Nebenwirkungen bei Immuntherapien. Trotz der hohen Prävalenz und klinischen Relevanz der immunvermittelten Sickness-Symptome sind Erwartungseffekte auf diese Symptome im spezifischen Kontext systemischer Entzündungsprozesse bisher kaum untersucht worden. Anhaltspunkte bieten in erster Linie Daten aus den Placebo-Gruppen randomisierter kontrollierter Medikamentenstudien bei chronisch-entzündlichen Erkrankungen wie rheumatoider Arthritis, bei Asthma, atopischer Dermatitis oder Allergien [13]. Die teils deutlichen Verbesserungen von entzündungsassoziierten Symptomen wie Schmerz oder Juckreiz in diesen Patient:innengruppen lassen auf Effekte von positiven Erwartungen auf immunvermittelte Symptome schließen. Darüber hinaus gibt es erste Hinweise, dass die Wirkung einer lokalen entzündungshemmenden Therapie bei Psoriasis durch positive Erwartungen verstärkt werden kann [1]. Auch bei Patient:innen mit atopischer Dermatitis ließen sich die Effekte einer intravenösen Infusion mit dem Antihistaminikum Dimetinden signifikant durch Erwartungen verbessern [27]. So führte die alleinige Erwartung, eine Anti-Juckreiz-Infusion zu erhalten, zu einer signifikant höheren Reduktion von experimentell erzeugtem Juckreiz und Quaddeln als eine verdeckte (das heißt für die Patient:innen unwissentlich verabreichte) Dimetinden-Infusion. Auch weisen erste Studien darauf hin, dass sich durch positive und negative Erwartungen die Nebenwirkungen einer Impfung beeinflussen lassen [20]. Somit ist es denkbar, durch die gezielte Induktion von Behandlungserwartungen die Wirksamkeit bzw. die Nebenwirkungen von immunmodulierenden Therapien zu beeinflussen und die Behandlung zu optimieren.

## Welche Mechanismen können Erwartungseffekte auf die Symptome des Sickness Behavior erklären?

Ein besseres Verständnis darüber, wie positive bzw. negative Erwartungen immunvermittelte Sickness-Symptome beeinflussen, kann dazu beitragen, psychologische Interventionen gezielt zur Erwartungsoptimierung, etwa im Kontext der Kommunikation mit Patient:innen, einzusetzen.

Das einführend beschriebene Endotoxinmodell wurde in zwei Studien genutzt, um Erwartungseffekte auf die immunvermittelten Sickness-Symptome in einem kontrollierten experimentellen Setting zu untersuchen. In der Studie einer schwedischen Arbeitsgruppe wurden Testpersonen im Vorfeld der Entzündungsinduktion nach ihren Erwartungen an den Verlauf der psychischen und körperlichen Krankheitssymptome nach LPS-Gabe gefragt [18]. Dabei wurde die erwartete Symptomausprägung im Kontrast zu bisher im Krankheitsfall erlebten Symptomen erfragt. Hierbei ergab sich, dass die erwarteten Symptome signifikant mit dem Ausmaß der tatsächlich erlebten negativen Emotionen nach LPS-Gabe assoziiert waren, jedoch nicht mit den körperlichen Symptomen des Sickness Behavior. So zeigten diejenigen Testpersonen, welche eine besonders geringe Symptomintensität erwarteten, nach der LPS-Gabe stärkere emotionsbezogene Symptome. Dies weist darauf hin, dass vermutlich insbesondere die emotionsbezogenen Sickness-Symptome durch Erwartungen an den Verlauf der Symptome gesteuert werden und ein „Mismatch“ zwischen erwarteten und erlebten Symptomen zu einem stärker ausgeprägten Sickness Behavior führt [18].

In einer weiteren Studie unserer Arbeitsgruppe wurden Daten aus den Kontrollgruppen von Endotoxinstudien analysiert [6]. Bei allen Testpersonen wurde nach Abschluss des Experimentaltages die wahrgenommene Gruppenallokation erfragt. Von den ca. 100 Testpersonen der Kontrollgruppen gaben nach Abschluss des Studientages ca. 20 Personen fälschlicherweise an, Endotoxin erhalten zu haben – obwohl faktisch eine wirkstofffreie Kochsalzlösung appliziert wurde. Diese Testpersonen („Nocebo-Responder“) berichteten über signifikant mehr und stärker ausgeprägte Sickness-Symptome als diejenigen, welche sich selbst korrekt der Kontrollgruppe zuordneten. Das Ausmaß der Sickness-Symptome war unter den „Nocebo-Respondern“ mit einer höheren Zustandsangst assoziiert, welche potenziell zu einer erhöhten Symptomaufmerksamkeit und damit zu den vermehrten Sickness-Symptomen beigetragen hat. Die Bedeutung von negativen Emotionen für die Entwicklung bzw. Verstärkung von Sickness-Symptomen wird auch durch die oben dargestellte Assoziation von entzündungsinduziertem Schmerz und negativer Stimmung im Endotoxinmodell gestützt. Eine relevante Frage für zukünftige Forschung ist, ob und inwieweit sich die Effekte negativer Emotionen durch die gezielte Induktion von Erwartungen beeinflussen lassen. Bedeutsam ist hierbei die Unterscheidung zwischen negativen Emotionen, die auf eine anstehende Behandlung gerichtet sind (z. B. Angst vor einem operativen Eingriff), und negativen Emotionen, die auf das Behandlungsergebnis (Angst vor Entzündungsschmerzen nach der Operation) gerichtet sind (vgl. Artikel von Stuhlreyer et al. in diesem Heft). So könnte eine behandlungsbezogene Angst (kurzfristige Angst vor einer zwar schmerzhaften, aber wirksamen Behandlung) potenziell sogar das Behandlungsergebnis verbessern und in Kauf genommen werden, da sie die Behandlung in ihrer Bedeutung aufwertet [26].

Aus der Placebo- und Noceboforschung ist inzwischen gut belegt, dass die Effekte von positiven und negativen Erwartungen auf Schmerz und affektbezogene Symptome über verschiedene biologische Systeme vermittelt werden, darunter das endogene Opioid- und Cholecystokininsystem sowie cannabinoide und dopaminerge Pfade [24]. Somit stellt sich die Frage, ob durch Erwartungen grundsätzlich auch die immunbiologischen Kommunikationswege beeinflusst werden können, über welche periphere Entzündungssignale das ZNS erreichen und somit Sickness Behavior auslösen. Zu diesen Kommunikationswegen zählt unter anderem die zytokinvermittelte Aktivierung des afferenten Vagusnervs (weiterführend [24]). Dadurch werden über den Nucleus tractus soletarii verschiedene Gehirnregionen beeinflusst, welche an der Ausbildung des Sickness Behavior beteiligt sind [10, 19]. Ein weiterer, unabhängiger Kommunikationsweg besteht in der Aktivierung von Gliazellen im ZNS, unter anderem durch proinflammatorische Zytokine und Prostaglandine [25, 30]. Zudem scheint die zentrale Prostaglandinsynthese dopaminerge Zellen im Striatum und in der Folge motivationale Prozesse während einer Immunaktivierung zu beeinflussen, die im Zusammenhang mit dem Sickness Behavior stehen [11]. Interessanterweise konnte im Mausmodell gezeigt werden, dass eine Aktivierung des (dopaminergen) Belohnungssystems, welches auch zur Ausbildung von Placeboeffekten beiträgt [24], die antibakterielle Immunantwort verstärkt [3].

Hinweise, dass Erwartungsprozesse proinflammatorische Mediatoren beeinflussen können, lassen sich aus der „PsyHeart-Studie“ ableiten, einer randomisierten kontrollierten Studie zu Erwartungseffekten im Kontext einer Bypass-Operation: Patient:innen, die präoperativ eine psychologische Intervention zur Erwartungsoptimierung im Hinblick auf das Ergebnis der Operation erhielten, wiesen sechs Monate nach der Operation – neben höherer psychischer Lebensqualität und subjektiver Belastbarkeit – niedrigere Plasmaspiegel proinflammatorischer Zytokine gegenüber einer Vergleichsgruppe auf [22]. Inwieweit die reduzierten Zytokinspiegel ein direktes Resultat der Erwartungsintervention waren oder vielmehr im Zusammenhang mit einem geringeren Distress stehen, bedarf jedoch weiterer Klärung. Erwartungseffekte auf Prostaglandinspiegel als einen weiteren Mediator des Sickness Behavior lassen sich aus einer Studie zum Höhenkopfschmerz ableiten [4]. So hatten diejenigen Testpersonen, die einen starken Höhenkopfschmerz erwarteten, nach dem Aufstieg auf 3500 m über dem Meeresspiegel in der Tat eine höhere Kopfschmerzinzidenz und Schmerzbelastung, welche mit höheren Prostaglandin E_2_-Konzentrationen assoziiert waren. Erhielten die Testpersonen eine wirkstofffreie Placebotablette, nahmen Prostaglandinspiegel und Kopfschmerzen ab.

Während diese Befunde zumindest indirekt darauf schließen lassen, dass Erwartungsprozesse auch die immunbiologischen Mechanismen des Sickness Behavior beeinflussen könnten, konnte in den beiden zuvor vorgestellten Endotoxinstudien [6, 18] kein Zusammenhang zwischen Erwartungen, Immunmediatoren und immunvermittelten Sickness-Symptomen gefunden werden. Somit ist eine weitere Untersuchung von Erwartungseffekten auf psychologische und immunbiologische Parameter im Kontext des Sickness Behavior notwendig, um ein weiterführendes Verständnis der Möglichkeiten (und Grenzen) einer psychologischen Beeinflussung von Symptomen des Sickness Behavior und auch der Wirksamkeit und der Nebenwirkungen von immunassoziierten Therapien durch Erwartungsinterventionen zu erreichen. Dies ist aktuell auch Gegenstand eines Teilprojekts im TRR-SFB 289 (www.treatment-expectation.de). Trotz der noch unvollständigen Befundlage lassen sich aus den oben dargestellten Studien sowie aus der weiterführenden Literatur (s. auch die weiteren Beiträge in diesem Heft) die nachfolgend dargestellten Hinweise ableiten, wie sich Erwartungseffekte in der Interaktion mit Patient:innen im Kontext des immunvermittelten Sickness Behavior berücksichtigen und nutzen lassen, um die Effekte von immunmodulierenden Medikamenten zu verstärken.

## Fazit für die Praxis

- Systemische Entzündungsprozesse gehen mit körperlichen und psychischen Krankheitssymptomen einher, darunter Schmerz und affektbezogene Symptome.
- Symptome des Sickness Behavior können im Kontext chronischer Entzündungsprozesse und als Nebenwirkungen von Immuntherapien eine erhebliche Belastung für die Betroffenen bedeuten.
- Die Vermittlung von grundlegendem Wissen über die Mechanismen und das Erscheinungsbild des immunvermittelten Sickness Behavior kann Betroffenen helfen, Symptome im Kontext systemischer Entzündungsprozesse besser einzuordnen, zu verstehen und zu bewältigen.
- Dabei sollten möglichst realistische Erwartungen an das Auftreten immunvermittelter Symptome entstehen, damit diese nicht durch ein „Mismatch“ verstärkt werden.
- Die Effekte von positiven und negativen Erwartungen auf schmerz- und stimmungsassoziierte Symptome sowie auf Wirkungen (und Nebenwirkungen) von Therapien sind umfassend belegt.
- Erwartungsprozesse können vermutlich auch zur Modulation von Symptomen des immunvermittelten Sickness Behavior beitragen und sollten in der Kommunikation mit Patient:innen berücksichtigt werden.
